# Improvement of esophageal cancer survival in Northeast Iran: A two-decade journey in a high-risk, low- resource region

**DOI:** 10.1371/journal.pone.0310842

**Published:** 2024-09-25

**Authors:** Saeed Nemati, Farhad Islami, Farin Kamangar, Hossein Poustchi, Gholamreza Roshandel, Ramin Shakeri, Allison Domingues, Masoud Khoshnia, Abdolsamad Gharavi, Paul Brennan, Christian C. Abnet, Sanford M. Dawsey, Paolo Boffetta, Reza Malekzadeh, Mahdi Sheikh

**Affiliations:** 1 Cancer Research Center, Cancer Institute, Tehran University of Medical Sciences, Tehran, Iran; 2 Genomic Epidemiology Branch, International Agency for Research on Cancer (IARC—WHO), Lyon, France; 3 Surveillance and Health Services Equity Research, American Cancer Society, Atlanta, GA, United States of America; 4 Department of Biology, School of Computer, Mathematical, and Natural Sciences, Morgan State University, Baltimore, MD, United States of America; 5 Liver and Pancreatobiliary Diseases Research Center, Digestive Diseases Research Institute, Tehran University of Medical Sciences, Tehran, Iran; 6 Golestan Research Center of Gastroenterology and Hepatology, Golestan University of Medical Sciences, Gorgan, Iran; 7 Digestive Oncology Research Center, Digestive Diseases Research Institute, Tehran University of Medical Sciences, Tehran, Iran; 8 Metabolic Epidemiology Branch, Division of Cancer Epidemiology and Genetics, National Cancer Institute, Bethesda, MD, United States of America; 9 Stony Brook Cancer Center, Stony Brook University, Stony Brook, NY, United States of America; 10 Department of Medical and Surgical Sciences, University of Bologna, Bologna, Italy; 11 Digestive Diseases Research Center, Digestive Diseases Research Institute, Tehran University of Medical Sciences, Tehran, Iran; Kermanshah University of Medical Sciences, ISLAMIC REPUBLIC OF IRAN

## Abstract

**Background and objective:**

Two decades ago, an international initiative (GEMINI) was launched in a high-risk, low-resource region in Northeast Iran, aiming to investigate incidence, etiology, early detection, and treatment of esophageal squamous cell carcinoma (ESCC). An earlier report from this area, highlighted poor ESCC survival rates, with a 5-year survival probability of 3.3% and the median survival time of 7 months. Our study assesses whether ESCC survival has improved since the implementation of the GEMINI initiative in this region.

**Material and methods:**

490 adult patients with histologically-confirmed ESCC were recruited from the Atrak clinic, Golestan, Iran, between 2007 and 2018. At recruitment, information on demographics and various exposures were collected. Active (telephone surveys) and passive (linkage to Golestan population-based cancer and death registries) follow-up methods were used to determine patients’ vital status though March 2019. Survival estimates were obtained by Kaplan-Meier method and Cox proportional hazards regression models.

**Results:**

Over the study period 340 deaths were recorded. Five-year ESCC survival probability was 23% (95% Confidence Interval: 19% to 28%), and the median survival time was 19 months. Five-year survival probability was higher among individuals who were younger (35% in <60-year-olds vs. 12% for >70-year-olds, p<0.001), educated (34% vs. 21% for no formal education, p = 0.027), never used opium (28% vs. 15%, p = 0.0016), and received cancer treatment (37% vs. 4%, p<0.001). In the adjusted models, a higher hazard of death was associated with older age [HR for each 10-year increase = 1.36 (95% CI = 1.22 to 1.51)], Turkman ethnicity [HR = 1.35 (95%CI: 1.07 to 1.70)], opium use [HR = 1.53 (95%CI: 1.20 to 1.94)],and receiving no cancer treatment [HR = 5.81 (95%CI: 3.97 to 8.52)].

**Conclusion:**

Over the last two decades, ESCC survival in this population has significantly improved, highlighting the potential of enhancing healthcare infrastructure and ensuring access to affordable medical care in resource-limited, high-risk regions. Older age at diagnosis, Turkman ethnicity, opium use, and untreated cases (indicative of advanced disease at diagnosis) were identified as the main ESCC prognostic factors in this population.

## Introduction

Golestan province in Northeast Iran has long been known for having one of the highest reported incidence rates of esophageal cancer worldwide [1]. The striking rates of esophageal cancer in this region led scientists from Tehran University of Medical Sciences, International Agency for Research on Cancer (IARC), and the US National Cancer Institute (NCI) to begin a series of investigations to identify the underlying etiologies and to monitor the incidence and mortality trends of esophageal cancer in this region [2]. This collaboration, referred to as “Gastro-Esophageal Malignancies in Northern Iran (GEMINI)”, also aimed to improve early detection, diagnosis, and treatment of upper gastrointestinal cancers in this high-risk population [3]. For this purpose, the Atrak Clinic (a referral gastroenterology specialized clinic) was established in the Golestan province to improve gastrointestinal health care services for the surrounding populations and to establish permanent local base for research studies [3, 4].

In the past two decades, the GEMINI project expanded our understanding of esophageal squamous cell carcinoma (ESCC), by thoroughly investigating risk factors that had been previously overlooked such as opium consumption [5], hot tea [6], and oral health [7]. In this specific region, GEMINI project resulted in identifying seven local risk factors that were estimated to contribute to 76% of ESCC cases in northeast Iran [2]. Also, GEMINI collaboration documented that the trend of esophageal cancer incidence has been continuously declining over the past decades in this region [8]. However, there is still a gap in knowledge about survival of patients diagnosed with esophageal cancer in this high-risk low-resource region in northeast Iran during the past decades. A previous study of ESCC cases diagnosed between 2002 and 2007, reported a very poor survival in this region, with a median survival time of 7 months and a 5-year survival probability of 3.3% [4]. By comparison, the 5-year survival probability for esophageal cancer in provinces with the highest development index in Iran, as well as in high-income countries such as the United States were reported at 20%-30% [9, 10].

For this analysis, we prospectively followed 490 patients with ESCC who were admitted to the Atrak clinic between 2007–2018, to investigate whether ESCC survival has improved since the implementation of the GEMINI initiative and the subsequent enhancements in diagnostic and therapeutic interventions provided by the establishment of this clinic in a low-resource region of Iran. We also assessed the prognostic factors related to ESCC survival in this population.

## Material and methods

### Study design and participants

This prospective cohort study involved 490 patients referred to the Atrak clinic in the Golestan region, Northeast Iran, who were diagnosed with ESCC, between 2007 and 2018. GEMINI initiative covered rural and urban areas of the Gonbad, Maraveh-tappeh, Kalaleh and Aq-qala districts of the Golestan province. Physicians in the study area were asked to refer all patients suspected of having upper gastrointestinal tract cancer to the Atrak Clinic, which was the only gastroenterology specialized clinic in Eastern Golestan. In the Atrak clinic, patients with suspicion of having upper gastrointestinal cancer underwent esophagogastroduodenoscopy. Biopsies were taken using a standardized protocol which were then examined by experienced pathologists. Eligible patients who received a histologically confirmed diagnosis of ESCC were invited to participate in this study. Inclusion criteria were residing in the study area, having at least 18 years of age, having histologically confirmed primary ESCC, and not having any history of previous cancer. This study was approved by the Institutional Review Board of the Digestive Diseases Research Institute at Tehran University of Medical Sciences and the International Agency for Research on Cancer (IARC).

### Baseline and follow-up data collection

Upon recruitment, the participants provided a written informed consent and underwent an interview using a brief questionnaire to collect information on demographics, medical history, lifestyle factors, and various exposures. Participants who smoked at least one cigarette per week for a minimum duration of 6 months were categorized as ever smoker. Similarly, those who consumed opium at least once per week for a minimum of 6 months were categorized as ever opium user. To avoid bias from reverse causation, participants who had started using cigarettes or opium one year prior to diagnosis were considered as non-users.

The study area was a remote region with limited infrastructure and facilities that are required for stage ascertainment. Further, many patients refused further workup for staging outside the study field. Therefore, we could not collect reliable staging information for the participants in this study [4]. Considering that the choice between receiving cancer treatment (surgery/chemotherapy/radiotherapy) and no cancer treatment (only palliative care) in this population was predominantly influenced by the clinical severity/advancement of their cancer at diagnosis, for adjustment purposes we used an ever/never treatment variable as a proxy for tumor stage at diagnosis.

Active and passive follow-up methods were applied to ascertain the participants’ vital status and treatment procedures. Participants were followed up annually by telephone to record information on their vital status and treatment procedures. Further, all relevant medical records were reviewed regularly by expert physicians to update the clinical and therapeutic information for each participant. To enhance the comprehensiveness of follow-up information, participants’ records were also matched to the Golestan population-based cancer registry [11], and the national death registry to confirm their vital status.

### Statistical analysis

We assessed the survival probabilities using the Kaplan-Meier method, and applied log-rank tests to evaluate the differences in survival estimates across the study subgroups. Cox proportional hazards regression models were used to estimate hazard ratios (HRs) and 95% confidence intervals (CIs) for assessing the prognostic effects of various demographical and clinical factors and different exposures on the survival of ESCC patients. For these models, entry time was defined as the date of ESCC diagnosis, and the exit time was set at the end of follow-up time, defined as the death date for those patients who died during the follow-up, or date of the last follow-up time for those who were still alive through the last follow-up on March 2019.

We investigated whether demographics and clinical factors at diagnosis could have any prognostic values among ESCC patients in this population using a minimally (age- and sex-) adjusted model, and a more extensively adjusted model that included age, sex, ethnicity, education, place of residence (urban, rural), tobacco use (cigarettes/waterpipe/chewing tobacco), opium use (ever/never), body mass index (BMI), and receiving any cancer treatment (no/yes). The proportional hazards (PH) assumption was tested using Schoenfeld’s global test. The treatment variable was the only variable that violated the PH assumption and therefore it was fitted as time-varying variable in the model.

Missing data were handled using Multiple Imputation [12]. Briefly, we imputed missing key variables using predictive mean matching multiple imputation under the assumption that variables were missing at random. Imputation was performed on ethnicity (Turkman vs non-Turkman), sex, residence (urban vs rural), cigarette smoking status, hookah use, nass (chewing tobacco) use, opium use, alcohol consumption, and BMI. These same imputed variables as well as vital status, age at diagnosis, year of diagnosis, family history of cancer, and survival days were included as predictors in the imputation set. We generated 15 imputed datasets using predictive mean matching. Multiple imputation was conducted using the MICE (Multivariate Imputation by Chained Equations) R package (version 3.16.0). We performed a sensitivity analysis to compare the estimates from the analyses using the complete vs. imputed datasets.

All statistical analyses were two-sided and performed using R and Stata software (StataCorp, Ver 17.0, College Station, Texas, USA). P-values less than 0.05 were considered statistically significant.

## Results

Of the 588 ESCC patients who were eligible for this study, 98 (16.6%) were excluded because they could not be tracked down to ascertain their vital status. The remaining 490 patients were included in this analysis. Demographic factors and various exposures were generally comparable among the excluded and included individuals in this analysis (**S1 Table**). At diagnosis, the mean (standard deviation) age of study participants was 64 (11.6) years, and for BMI was 22.0 (4.7) kg/m^2^. Of the included participants, 257 (52%) were male, and 287 (60%) were of Turkman ethnicity. Most participants had no formal education (n = 414, 87%) and were residing in rural areas (n = 360, 75%). Ever tobacco use was reported by 113 participants (25%), and 169 participants (37%) reported ever use of opium. Only 2 participants reported ever alcohol consumption. Notably, 131 participants (27%) reported a positive family history for esophageal cancer among their first-degree relatives. Of the included participants, 55% received treatment (surgery / chemotherapy / radiotherapy) for their cancer (**Table 1**). The proportion of patients who received treatment for ESCC constantly increased over time (20% of patients who were diagnosed in 2009 and earlier vs. 80% of those who were diagnosed in 2016 onwards) (**S1 Fig**). Similarly, the proportion of patients who had higher BMI at diagnosis increased over time (18% of patients who were diagnosed in 2009 and earlier were overweight vs. 30% of those who were diagnosed in 2016 onwards).

**Table 1:** Estimates of survival rates by demographical features and various exposures among patients with esophageal squamous cell carcinoma who were diagnosed between 2007–2018 in the Atrak clinic in northeast Iran.

Characteristics	N of failure/N (%) ^1^	Median survival time (months)	1 year survival	3 years survival	5 years survival	P-value ^2^
**Total**	340/490 (69%)	19	63%	32%	23%	
**Age (years)**						**< 0.001**
	100/168 (59%)	30	72%	47%	35%	
60–70	115/166 (69%)	21	67%	32%	22%	
>70	125/156 (80%)	12	49%	15%	12%	
**Sex**						0.086
Male	195/257 (76%)	16	60%	30%	20%	
Female	145/233 (62%)	20	67%	34%	27%	
**Ethnicity**						0.076
Non-Turkman	132/196 (67%)	22	68%	33%	27%	
Turkman	203/287 (71%)	14	60%	31%	21%	
**Formal education**						**0.027**
Yes	36/63 (57%)	34	77%	48%	34%	
No	297/414 (71%)	15	61%	29%	21%	
**Residence**						0.21
Urban	77/122 (61%)	22	70%	32%	26%	
Rural	262/360 (73%)	17	61%	31%	22%	
**Body mass index (BMI, kg/m**^**2**^)						**< 0.001**
Underweight (BMI<18)	55/67 (82%)	12	48%	25%	16%	
Normal (18≤BMI<25)	140/192 (73%)	18	62%	31%	22%	
Overweight/Obese (BMI≥25)	47/83 (57%)	36	81%	48%	40%	
**Opium use**						**0.0016**
Never	184/284 (65%)	22	66%	35%	28%	
Ever	133/169 (79%)	13	54%	26%	15%	
**Tobacco use**						0.34
Never	231/339 (68%)	19	63%	33%	24%	
Ever	85/113 (75%)	16	60%	28%	19%	
**Any cancer treatment** ^**3**^						**< 0.001**
Yes	139/267 (52%)	36	84%	51%	37%	
No	201/223 (90.1%)	10	44%	6%	4%	No

^**1**^ Of the total eligible participants: education is missing for 13; ethnicity is missing for 7; residence is missing for 8; body mass index is missing for 148; opium use status for 37; and tobacco use status for 38.

^**2**^ Log-rank test P value

^**3**^ Treatment includes undergoing surgery or chemotherapy or radiotherapy.

**Fig 1:**
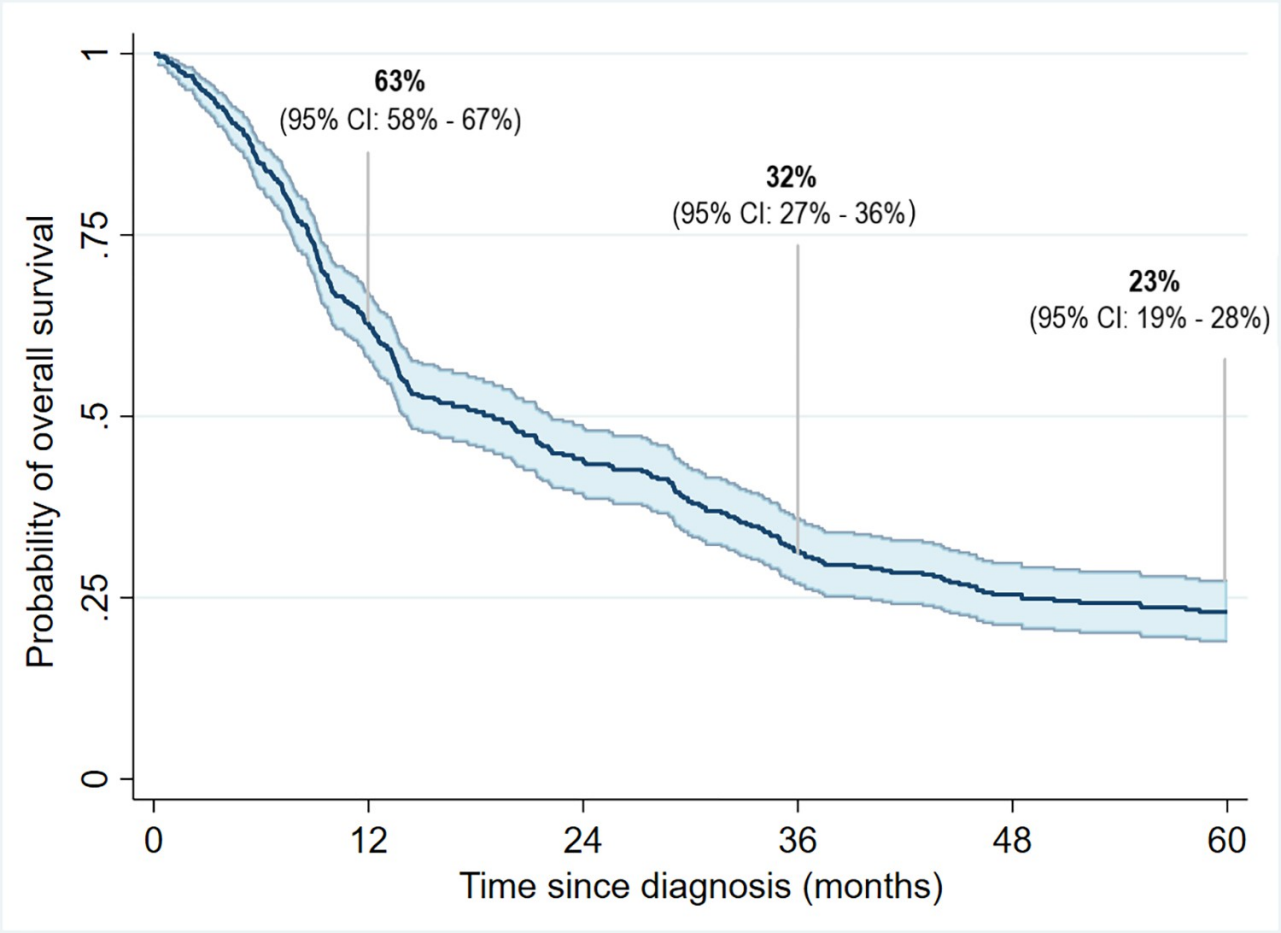
Overall survival in the first 5 years of diagnosis among patients with esophageal squamous cell carcinoma who were diagnosed between 2007–2018 in the Atrak clinic in northeast Iran.

**Fig 2:**
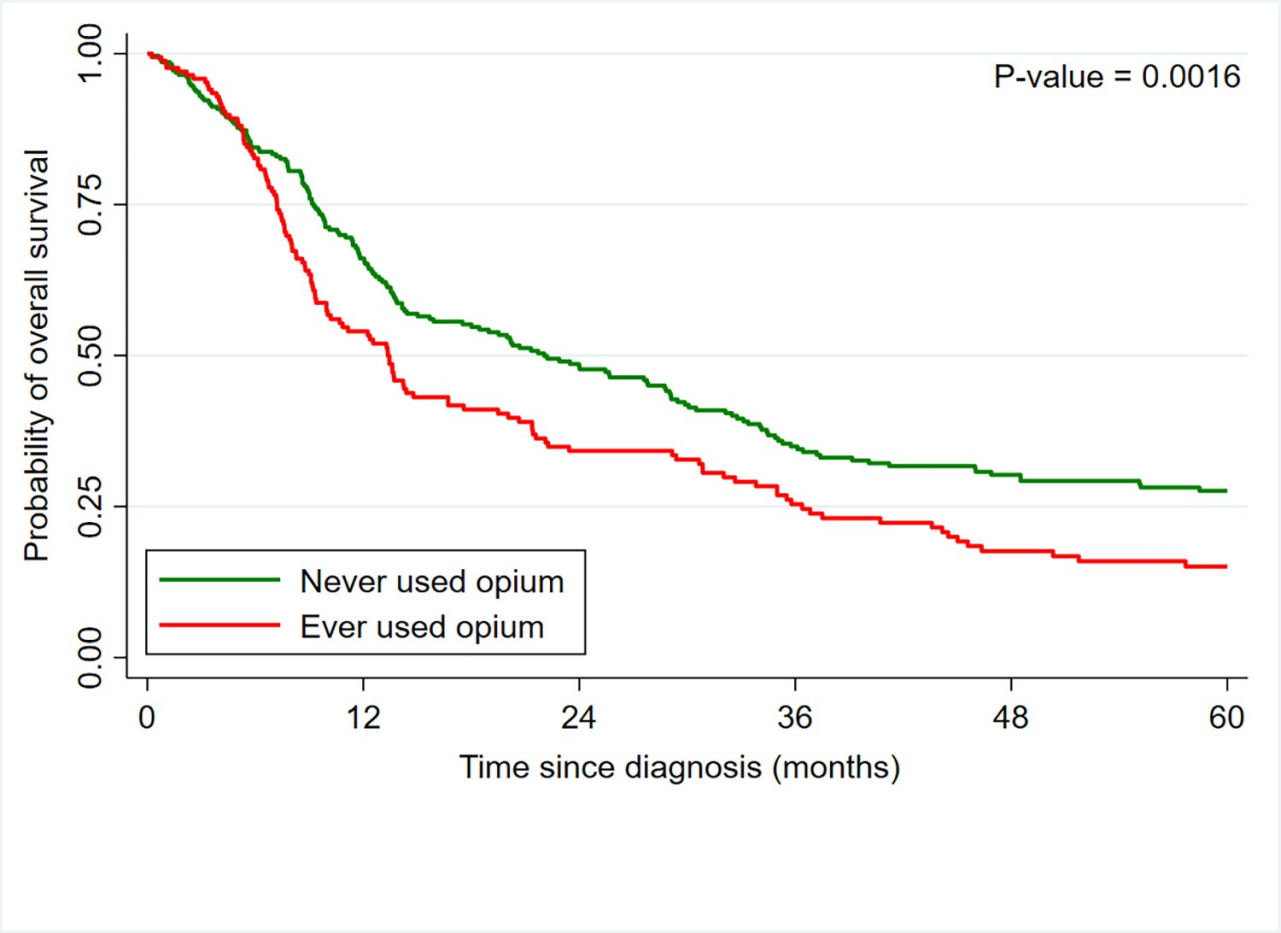
Overall survival by opium use status at baseline among patients with esophageal squamous cell carcinoma who were diagnosed between 2007–2018 in the Atrak clinic in northeast Iran.

Patients with ESCC had an overall 1-year survival probability of 63%, while the 3- and 5- year survival probabilities were 32% and 23%, respectively (**Fig 1**). The five-year survival probability was higher among individuals who were younger (5-year survival at 35% for ages <60 vs. 12% for ages >70 years, p<0.001), had formal education (34% vs. 21%, p = 0.027), higher BMI at diagnosis (40% for BMI≥25 vs. 16% for BMI<18 kg/m^2^, p<0.001), never used opium (28% vs. 15% among opium users, p = 0.0016, **Fig 2**), and were treated for ESCC (37% vs. 4% who did not receive any cancer treatment, p<0.001). Survival probabilities were comparable across the strata of sex, ethnicity, residence, and tobacco use (**Table 1**).

After adjusting for potential confounders and risk factors, an elevated hazard of death remained associated with older age [HR for each 10-year increase = 1.36 (95% CI: 1.22 to 1.51)], Turkman ethnicity [HR = 1.35 (95% CI: 1.07 to 1.70)], opium use [HR = 1.53 (95% CI: 1.20 to 1.94)] and receiving no treatment [HR = 5.81 (95% CI: 3.97 to 8.52)] (**Table 2**). The sensitivity analysis (complete case) yielded comparable results (**S2 Table**).

**Table 2:** Risk of death by baseline demographical and clinical features among patients with esophageal squamous cell carcinoma who were diagnosed between 2007–2018 in the Atrak clinic in northeast Iran.

Characteristic	N (%)	Age- and sex-adjusted HR (95% CI)	Fully adjusted Model ^1^ HR (95% CI)
**Age (years)**			
Each year increase in age	-	**1.36 (1.23–1.50)**	**1.36 (1.22–1.51)**
**Sex**			
Female	233 (48%)	1	1
Male	257 (52%)	1.15 (0.92–1.43)	1.06 (0.83–1.36)
**Ethnicity**			
Non-Turkman	199 (41%)	1	1
Turkman	297 (59%)	**1.27 (1.02–1.59)**	**1.35 (1.07–1.70)**
**Formal education**			
Yes	66 (13%)	1	1
No	424 (87%)	1.34 (0.93–1.94)	1.01 (0.69–1.50)
**Residence**			
Urban	122 (25%)	1	1
Rural	368 (75%)	1.22 (0.93–1.59)	1.21 (0.91–1.62)
**Body mass index (BMI, kg/m**^**2**^)			
Underweight (BMI<18)	97 (20%)	1	1
Normal (18≤BMI<25)	260 (53%)	0.84 (0.65–1.10)	1.09 (0.83–1.42)
Overweight/Obese (BMI≥25)	133 (27%)	**0.47 (0.34–0.65)**	0.75 (0.52–1.08)
**Opium use**			
Never	308 (63%)	1	1
Ever	182 (17%)	**1.53 (1.23–1.91)**	**1.53 (1.20–1.94)**
**Tobacco use**			
Never	360 (73%)	1	1
Ever	130 (27%)	1.19 (0.93–1.53)	0.88 (0.67–1.15)
**Any cancer treatment** ^**2**^			
Yes	267 (55%)	1	1
No	223 (45%)	**6.07 (4.17–8.84)**	**5.81 (3.97–8.52)**

^**1**^ Fully adjusted model includes all variables shown in this table.

^**2**^ Treatment includes undergoing surgery or chemotherapy or radiotherapy.

## Discussion

Following up on 490 adult patients diagnosed with ESCC in the past two decades in the high-risk region of northeast Iran showed overall 1- and 5-year survival probabilities of 63% and 23%, respectively, with a median survival time of 19 months. Older age at diagnosis, Turkmen ethnicity, opium use, and not receiving any treatment (as a proxy for advanced disease at diagnosis) were identified as the main ESCC prognostic factors in this population. Notably, Turkman ethnicity and opium use are among the main etiologic risk factors of ESCC identified in this population [2].

In comparison to a previous report that included patients diagnosed at the same clinic before 2007 (the initial years of GEMINI implementation) [4], our findings suggest a substantial increase in 5-year survival probability, rising from 3% to 23%, and a notable extension in median survival time, progressing from 7 to 19 months for ESCC cases diagnosed in 2007 and onward within the same clinic. Notably, this study, conducted in a high-risk remote region of Golestan province, reveals an esophageal cancer survival rate surpassing that reported for the entire Golestan province (5-year survival rate: 10%-15%) [13, 14]. Furthermore, the survival rates in this population reached those reported in provinces with the highest development index in Iran (5-year survival rate: 23%-27%) [9], as well as survival rates in high-income countries such as the United States and United Kingdom (20%-30%) [15–17]. Interestingly, after adjustment for treatment reception and other confounders, socioeconomic indicators such as education and urban/rural residence did not show any prognostic value in this population. Altogether, these findings underscore the potential impact of enhancing access to affordable high-quality medical care in reducing survival disparities among esophageal cancer patients across regions with varying levels of development and income indices.

In addition to providing access to affordable gastroenterology care, various factors may have contributed to the enhanced ESCC survival observed in this high-risk population over two decades of GEMINI initiative implementation. These factors include engaging officials at various levels to improve healthcare infrastructure and facilitate patient referral process in the region, and raising awareness among the primary healthcare providers and the public regarding ESCC and its risk factors (that may also affect survival such as opium use) [3]. While, due to logistical challenges, we could not collect stage information, the consistent rise in the proportion of ESCC patients receiving cancer treatment (from 20% in 2007–2009 to 80% in 2016–2018), along with the increase in the proportion of patients with higher BMI at diagnosis in this clinic, may suggest a continuous increase in the proportion of ESCC cases that are detected at earlier stages when patients are eligible for and can benefit from treatment [18].

Despite the observed improvement in ESCC survival within this population, the 5-year ESCC survival rate remains poor. This highlights the need for studies that investigate modifiable prognostic factors with the potential to reduce the burden of esophageal cancer by enhancing survival, particularly in high-risk populations [17]. Beyond the prognostic values of non-modifiable risk factors such as age and ethnicity, that are also documented in other populations [19–21], we found that the receipt of cancer treatment and using opium to be linked with ESCC prognosis in this population. In line with research in other high-risk regions [22], currently investigations are ongoing in this region to find feasible non-invasive approaches that may help early detection of ESCC and subsequently decreasing its burden in this region [23].

In the current study, the use of opium was linked to a more than 50% increased risk of death among ESCC patients. This finding is in line with our recent study of pancreatic cancer survival in Iran, where we also found that opium use was associated with a more than 50% increased risk of death [24]. Opium, a highly addictive narcotic derived from the poppy plant, is widely utilized in central and western Asia for recreational and pain-relieving purposes [25]. While the International Agency for Research on Cancer (IARC) recently classified opium consumption as carcinogenic to humans [26], our results suggest the hazards of opium consumption may extend beyond cancer diagnosis and impact the survival of cancer patients [24]. Similar to the impacts we observed regarding opium use on cancer survival, a retrospective study in South Korea revealed that chronic preadmission use of opioids was linked to an increased risk of death among cancer patients [27]. While it is plausible that the pain-relieving properties of opium and opioids, in general, could contribute to delayed referral, resulting in higher tumor stages at diagnosis with poorer prognosis [28], the significant association of opium use with an elevated risk of death among cancer survivors, even after thorough adjustment for tumor stage and/or treatments [24], suggests a possible direct impact of opium on the survival of cancer patients who use it. Experimental studies have indicated that opioids may promote tumor growth, invasiveness, and metastasis, providing a potential explanation for the higher death risk among opium users [29]. Other proposed mechanisms may involve the detrimental effects of opium use on various organs, such as cardiovascular and respiratory systems [30–32].

This study represents a unique example, illustrating how improvements in healthcare infrastructure and the provision of affordable, high-quality medical care in a resource-limited remote region, coupled with heightened awareness among the public and healthcare workers in a high-risk area, can markedly enhance cancer survival, bringing them in line with those observed in high-income countries. This is in line with studies conducted among disadvantaged patients in high-income countries, which showed how implementation of access to high-level diagnostic and treatment facilities improved cancer survival [33].

Other strengths of this study include its prospective design, long follow up duration, large sample size, and being the first study that demonstrated the detrimental effects of opium consumption, that is widely used for pain relieving purposes in this region, on the survival of esophageal cancer patients. However, this study also has several limitations. The primary limitation is the lack of information on tumor stage at diagnosis. Although we tried to adjust for receiving cancer treatment as a proxy for disease severity in our models, we cannot rule out the possibility that the cancer treatments received by the patients may not completely adhere to guidelines. Consequently, residual confounding stemming from higher stage at diagnosis may partly account for some observed associations in the adjusted models, including the link between opium use and poorer survival. Despite this limitation, the consistency and similarity of risk estimates for mortality related to opium use in both minimally adjusted and fully adjusted models (HR = 1.53 in both) make it unlikely that residual confounding entirely explains the observed associations. An additional limitation was lacking data on the duration and frequency of tobacco and opium use. Collecting such information could have facilitated dose-response analyses, providing a deeper understanding of the impact of these substances on ESCC survival.

In conclusion, the two decades of implementing the GEMINI initiative in the high-risk region of Northeast Iran coincided with and likely contributed to a substantial improvement in ESCC survival within this population. This study can serve as an exemplary demonstration that ESCC survival in high risk and resource-limited can reach levels comparable to those in high-income countries through raising awareness among the public and healthcare providers, enhancing healthcare infrastructure, and ensuring access to affordable, high-quality medical care. Additionally, we found that ESCC patients who were older, belonged to the Turkman ethnicity, and those who used opium had an increased risk of experiencing a poorer prognosis.

## Supporting information

S1 Checklist STROBE statement—Checklist of items that should be included in reports of *cohort studies*. (DOCX)

S1 Table Comparison between eligible participants who were lost to follow-up (excluded) and those who were successfully followed (included) in this analysis. (DOCX)

S2 Table Complete case analysis (n = 331) investigating risk of death by baseline demographical and clinical features among patients with esophageal squamous cell carcinoma who were diagnosed between 2007–2018 in the Atrak clinic in northeast Iran. (DOCX)

S1 Fig The proportion of esophageal squamous cell carcinoma patients who received cancer treatment (surgery, chemotherapy, radiotherapy) during the study period. (PDF)
