# Supplementary material for: Improvement of esophageal cancer survival in Northeast Iran: A two-decade journey in a high-risk, low- resource region
Source: PLoS One. 2024 Sep 25;19(9):e0310842. doi: 10.1371/journal.pone.0310842 (PMC11423987; doi:10.1371/journal.pone.0310842)
Supplement: S1 Table — (DOCX) [file pone.0310842.s002.docx]

**S1 Table.** Comparison between eligible participants who were lost to follow-up (excluded) and those who were successfully followed (included) in this analysis.

| **Characteristics** | **Included Eligible Participants (n=490)** | **Excluded Eligible Participants (n=98)** | **P-value** |
| --- | --- | --- | --- |
| **Age (mean (± SD)), years** | 64 (±11.6) | 66 (±10.2) | 0.11 |
| **Sex, n (%)** |  |  | 0.45 |
| Female | 230 (47%) | 42 (43%) |  |
| Male | 260 (53%) | 56 (58%) |  |
| **Ethnicity, n (%) *** |  |  | 0.97 |
| Non-Turkman | 40 (41%) | 198 (41%) |  |
| Turkmen | 58 (59%) | 285 (59%) |  |
| **BMI (Kg/m^2^), Mean (SD)** | 22 (±0.2) | 21 (±0.4) | 0.08 |
| **Place of residence, n (%)** |  |  | 0.7 |
| Rural | 361 (75%) | 71 (73%) |  |
| Urban | 120 (25%) | 26 (27%) |  |
| **Formal education, n (%)** |  |  | 0.18 |
| No | 414 (87%) | 88 (92%) |  |
| Yes | 63 (13%) | 8 (8%) |  |
| **Tobacco status, n (%)** |  |  | **0.03** |
| Non-user | 338 (75%) | 59 (64%) |  |
| User | 113 (25%) | 33 (36%) |  |
| **Opium use, n (%)** |  |  | 0.46 |
| No | 319 (65%) | 60 (61%) |  |
| Yes | 171 (35%) | 38 (39%) |  |

**SD:** Standard Deviation

* Of the total eligible participants: ethnicity is missing for 7; residence is missing for 10; education is missing for 15; tobacco use status for 45; opium use status for 44.
