# Supplementary material for: Improvement of esophageal cancer survival in Northeast Iran: A two-decade journey in a high-risk, low- resource region
Source: PLoS One. 2024 Sep 25;19(9):e0310842. doi: 10.1371/journal.pone.0310842 (PMC11423987; doi:10.1371/journal.pone.0310842)
Supplement: S2 Table — (DOCX) [file pone.0310842.s003.docx]

**S2 Table.** *Complete case analysis (n = 331)* investigating risk of death by baseline demographical and clinical features among patients with esophageal squamous cell carcinoma who were diagnosed between 2007 – 2018 in the Atrak clinic in northeast Iran.

| **Characteristic** | **Age- and sex-adjusted**  **HR (95% CI)** | **Fully adjusted Model ^1^**  **HR (95% CI)** |
| --- | --- | --- |
| **Age (years)** | |  |
| Each year increase in age | **1.36 (1.23 – 1.50)** | **1.39 (1.22 – 1.60)** |
| **Sex** | |  |
| Female | 1 | 1 |
| Male | 1.15 (0.92 – 1.43) | 1.07 (0.79 – 1.45) |
| **Ethnicity** | |  |
| Non-Turkman | 1 | 1 |
| Turkman | **1.30 (1.04 – 1.62)** | **1.32 (0.99 – 1.76)** |
| **Formal education** | |  |
| Yes | 1 | 1 |
| No | 1.34 (0.93 – 1.93) | 0.91 (0.57 – 1.43) |
| **Residence** | |  |
| Urban | 1 | 1 |
| Rural | 1.24 (0.95 – 1.61) | 0.90 (0.64 – 1.28) |
| **Body mass index (BMI, kg/m^2^)** | |  |
| Underweight (BMI<18) | 1 | 1 |
| Normal (18≤BMI<25) | 0.79 (0.57 – 1.07) | 1.00 (0.72 – 1.40) |
| Overweight/Obese (BMI≥25) | **0.43 (0.29 – 0.63)** | 0.68 (0.44 – 1.05) |
| **Opium use** | |  |
| Never | 1 | 1 |
| Ever | **1.49 (1.18 – 1.87)** | **1.43 (1.06 – 1.91)** |
| **Tobacco use** | |  |
| Never | 1 | 1 |
| Ever | 1.14 (0.87 – 1.48) | 0.89 (0.63 – 1.26) |
| **Any cancer treatment ^2^** | |  |
| Yes | 1 | 1 |
| No | **6.07 (4.17 – 8.84)** | **7.54 (4.62 – 12.28)** |

**^1^** Fully adjusted model includes all variables shown in this table.

**^2^** Treatment includes undergoing surgery or chemotherapy or radiotherapy.
