# Supplementary material for: Improvement of esophageal cancer survival in Northeast Iran: A two-decade journey in a high-risk, low- resource region
Source: PLoS One. 2024 Sep 25;19(9):e0310842. doi: 10.1371/journal.pone.0310842 (PMC11423987; doi:10.1371/journal.pone.0310842)
Supplement: S1 Fig — (PDF) [file pone.0310842.s004.pdf]

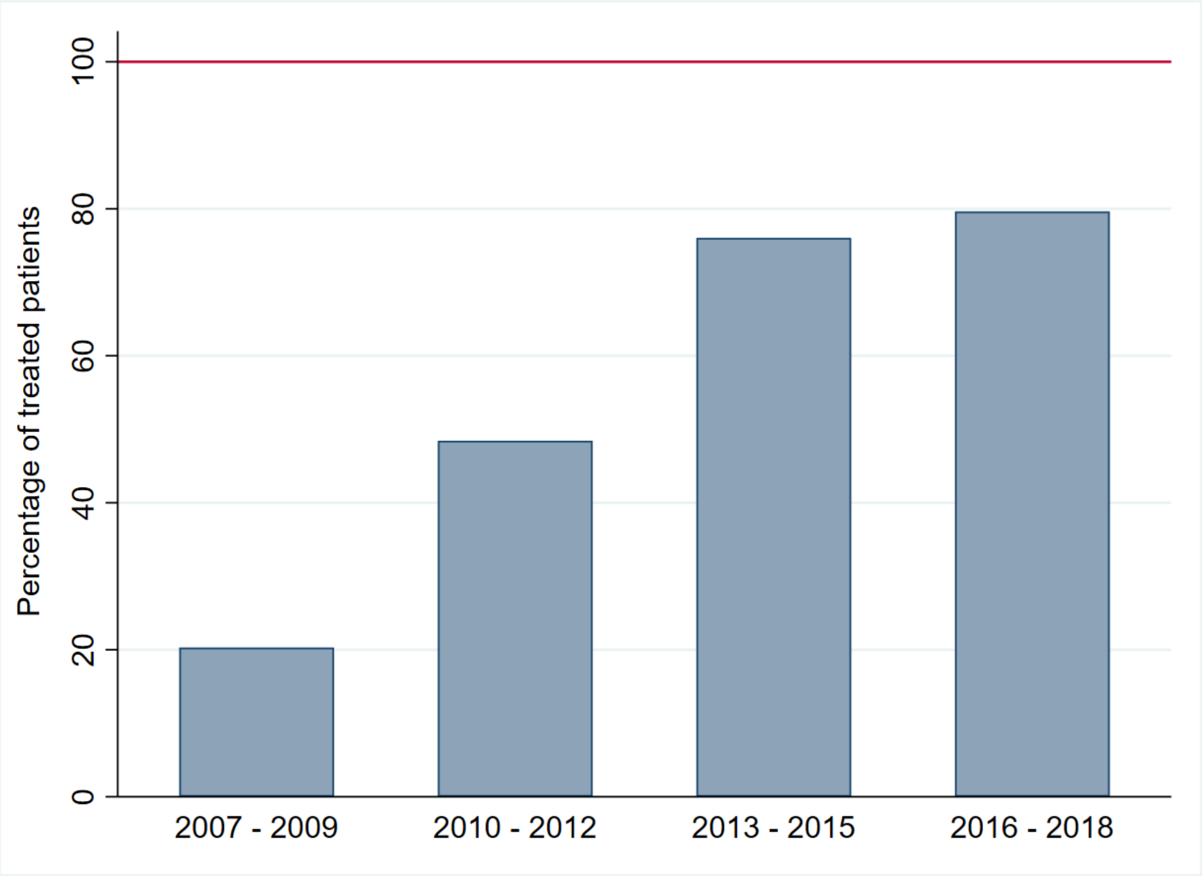

S1 Figure. The proportion of esophageal squamous cell carcinoma patients who received cancer treatment (surgery, chemotherapy, radiotherapy) during the study period.
